# Fetal alcohol spectrum disorder (FASD): how primary care can make a difference

**DOI:** 10.3399/BJGP.2025.0587

**Published:** 2025-12-01

**Authors:** Cheryl McQuire, Lou Millington, Amy Dillon, Andy Boyd, James Parsonage, Raja Mukherjee, Sandra I Butcher, Patricia D Jackson

**Affiliations:** 1 Bristol Medical School, University of Bristol, Bristol, UK; 2 NHS Bristol, North Somerset and South Gloucestershire Integrated Care Board (BNSSG ICB), Bristol, UK; 3 South Yorkshire Integrated Care Board (ICB), Sheffield, UK; 4 NIHR Bristol Biomedical Research Centre, University of Bristol, Bristol, UK; 5 Surrey and Borders Partnership NHS Foundation Trust, Surrey, UK; 6 University of Surrey, School of Medicine, Surrey, UK; 7 National Organisation for FASD, Hertfordshire, UK; 8 Past President and continuing member of Scottish Paediatric Society, Past Chair and continuing member Scottish Association of Community Child Health, London, UK; 9 Paediatric Representative Scottish Health Action on Alcohol Problems; sponsored by Royal College of Physicians of Edinburgh, Edinburgh, UK

Around 4% of the UK population are living with fetal alcohol spectrum disorder (FASD) because of prenatal alcohol exposure.^1,2^ For a typical GP practice of 10 000 patients this means at least 400 people with FASD per practice. Or, equivalently, this is one child in each class of 25–30 pupils.^3^ Among vulnerable groups, including people who have been through the care system, are homeless, have addiction problems, or interface regularly with the justice system, prevalence is much higher with studies suggesting 20–40% may be living with FASD.^4^ Despite its significant prevalence and the fact that early diagnosis and support can dramatically improve outcomes, FASD often goes unrecognised, especially in adults.

## What is fetal alcohol spectrum disorder?

FASD is a lifelong neurodevelopmental condition resulting from prenatal alcohol exposure. Diagnosis requires evidence of prenatal alcohol exposure and significant impairment in three or more of the following domains: motor skills, neuroanatomy or neurophysiology, cognition, language, academic achievement, memory, attention, executive function (including impulse control and hyperactivity), affect regulation, and adaptive behaviour, social skills, or social communication.^5,6^

Historically, diagnosis of FASD focused on three sentinel facial features — smooth philtrum, thin upper lip, and short palpebral fissures. When all three are present, they are so specific to prenatal alcohol exposure that confirmation of exposure through other sources (for example, reliable clinical observation, screening, self-report, alcohol treatment history) is not required. However, these facial features only occur in up to 10% of cases, making FASD largely an ‘invisible’ condition. Where sentinel facial features are absent, reliable evidence of maternal alcohol use during pregnancy is essential for diagnosis.^5,6^

With over 400 associated comorbidities spanning 18 of the 22 International Statistical Classification of Diseases and Related Health Problems, 10th Revision (ICD-10) chapters,^4^ primary care clinicians will be faced with FASD across the lifespan in multiple contexts. Keen awareness can help mitigate the disproportionate health and social impacts.

## Advances in national guidance and implications for GPs

Recent landmark guidance from the National Institute for Health and Care Excellence (NICE Quality Standard for FASD**,** QS04),^5^ Scottish Intercollegiate Guidelines Network (SIGN 156: *Children and Young People Exposed Prenatally to Alcohol*),^6^ and Department of Health and Social Care (DHSC Health Needs Assessment for FASD)^7^ provides clear, GP-relevant steps to identify and support people with suspected or confirmed FASD.

By following national guidance and reliably documenting diagnoses in electronic patient records, practices can improve individual patient care, generate accurate local prevalence data, and contribute to national research and commissioning. Those living with FASD tell us that having this diagnosis recognised in their records is crucial. Without it, people living with FASD have to repeatedly explain their condition, which can lead to frustrating and uncomfortable conversations. As one person with FASD explained in our recent knowledge mobilisation work:^8^

*‘I want them to note that I have fetal alcohol spectrum disorder (FASD) in my records. It won’t only just help me, it will help the other doctors as well to know to take things slower and explain things step by step.’*

Documented recognition ensures reasonable adjustments are made, care is tailored, and people with FASD feel ‘seen’.

## The role of GPs

GPs can do much to support FASD prevention, diagnosis, and management. Drawing on guidance from the Chief Medical Officers, DHSC, NICE, and SIGN,^5–7,9^ we have highlighted key areas of action for practice teams (Box 1).

Box 1. **Recommendations for the role of GPs in fetal alcohol spectrum disorder (FASD) prevention, diagnosis, clinical coding, and management**

- Give women and their supporters clear and consistent advice on avoiding alcohol throughout pregnancy and when planning pregnancy, consistent with Chief Medical Officers’ Guidance (2016).^9^
- Actively consider prenatal alcohol exposure as a possible underlying cause for neurodevelopmental delay, or unexplained departures from a typical developmental profile.^5,6^
- Refer people with probable prenatal alcohol exposure and significant physical, developmental, or behavioural difficulties for assessment^5,6^ (see ‘Guidance and referral pathways’ section below for more information).
- Ensure that anyone with FASD is offered a management plan to address their specific needs, and an annual disability check-up, paying particular attention to any outstanding medical issues.^5–7^
- GPs and practice teams should use the new SNOMED CT codes consistently for patients with confirmed or suspected FASD to document this condition in their electronic patient record (see ‘Coding is caring’ section below for details).

## Guidance and referral pathways

For children and young people, GPs need to know that referral for assessment is typically to community paediatrics, child development centres, or Child and Adolescent Mental Health Services, ideally by a healthcare professional with additional training in FASD.^5^ In the authors’ experience, if a referral is made specifically for an ‘FASD assessment’ where no FASD pathway exists, this can often be denied. However, if FASD is identified within the course of a wider neurodevelopmental assessment, diagnosis can usually proceed.

For adults with suspected FASD, the picture is more complex. The first step may be to request a neuropsychological assessment via a clinical psychologist or referral to a specialist FASD clinic, or other clinical services available through the FASD hub-and-spoke model. The proposed hub-and-spoke model places a specialist national/regional centre at the core, responsible for overseeing FASD services, managing the most complex cases, and offering expert clinical guidance (currently the national FASD clinic is within Surrey and Borders Partnership NHS Foundation Trust). Surrounding this, regional/local clinics would handle routine, more straight forward cases, referring patients onward to more specialist services when greater expertise is required.^7^

Of course, the authors recognise that assessment and care pathways for FASD remain inconsistent, complex, under-funded, and subject to the significant backlogs within neurodevelopmental services. However, this should not mean that people with suspected FASD are not put forward for assessment and support.^5–7^

## ‘Coding is caring’

The introduction of new SNOMED CT codes in 2024 means that, for the first time, the full spectrum of FASD can be properly recorded in electronic patient records.^8^

As highly trusted custodians of their patients’ data, GPs are aware that ‘coding is caring’.^10^ Accurate, consistent coding is not an administrative exercise but a cornerstone of safe, equitable, and effective patient care.

**Figure 1.**
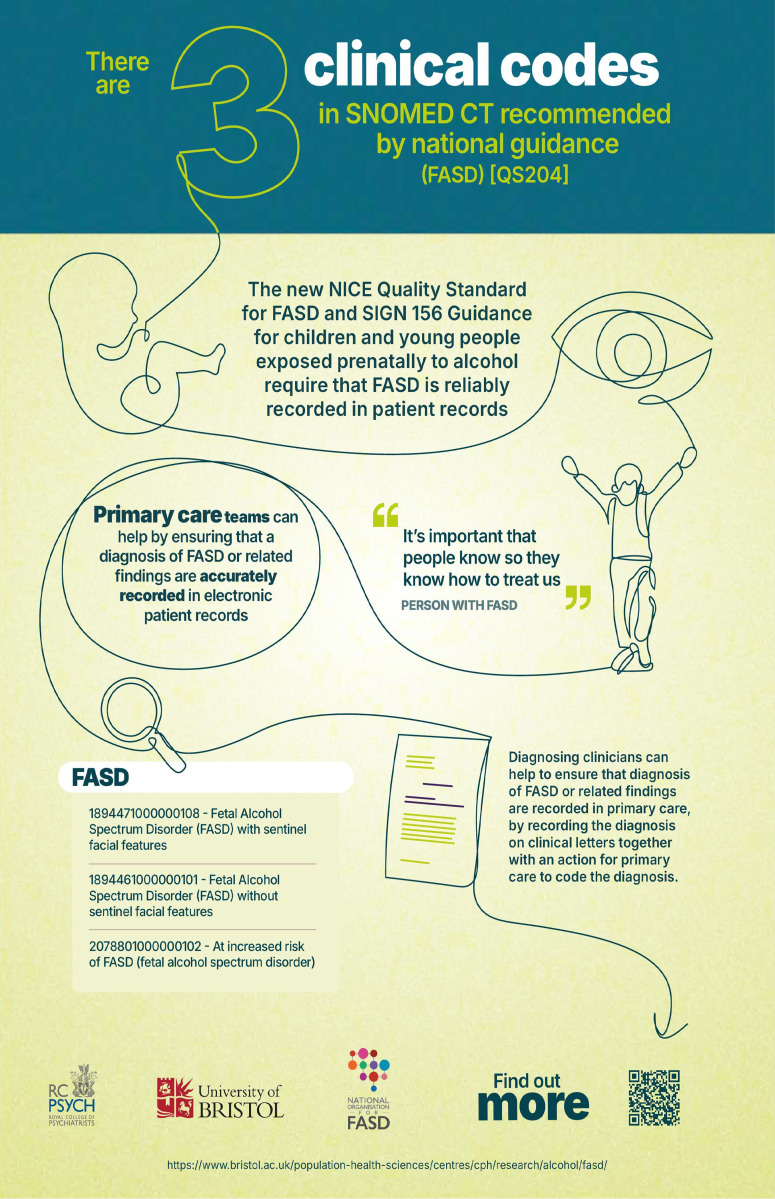
New SNOMED CT codes and clinical coding pathway for fetal alcohol spectrum disorder. All knowledge mobilisation materials, including infographics, 1-minute animation, and social media thumbnails, are freely and publicly available for download and sharing: https://osf.io/n2agk/files/osfstorage. They can also be accessed via the QR code in the figure.

FASD codes are now searchable to GPs in England within EMIS and SystmOne, with equivalent codes available in Scotland (Figure 1).^8^ They are 1) ‘Fetal Alcohol Spectrum Disorder with sentinel facial features’, 2) ‘Fetal Alcohol Spectrum Disorder without sentinel facial features’, and (3) ‘At increased risk of fetal alcohol spectrum disorder’.

By adopting these codes, practices can improve patient care and ensure that care teams are informed and able to make reasonable adjustments. For population health, accurate recording of FASD fills a longstanding evidence gap, enabling better surveillance, research, and service commissioning.^8^ This push for accurate FASD coding reflects broader NHS priorities. NHS England’s Data Saves Lives strategy,^11^ NHS 10 Year Health Plan,^12^ and the move towards ‘digital as default’ depend on robust, standardised data capture at the point of care. Clinical coding is no longer a back-office concern but central to quality, safety, and equity.

## A call to action: ‘Neglect of FASD must end’

We stand at a pivotal moment. National guidance is in place,^5–7^ the codes exist,^8^ and education and awareness is rising through campaigns such as International FASD Awareness Month (September) and workforce training. What is needed now is action to ensure that ‘neglect of FASD’^3^ ends once and for all.

### Further information and open access materials

Full details of the knowledge mobilisation project underpinning this editorial can be found on the project webpage: https://www.bristol.ac.uk/fasd-codes and associated blog ‘Recognising the hidden disability of FASD in electronic patient records’: https://population-health.bristol.ac.uk/2024/10/04/recognising-the-hidden-disability-of-fetal-alcohol-spectrum-disorder-fasd-in-electronic-patient-records/.

All knowledge mobilisation materials, including infographics, 1-minute animation and social media thumbnails, are freely and publicly available for download and sharing via the Open Science Framework (OSF) website: https://osf.io/n2agk/files and via the QR code in Figure 1.
